# Asperlin Stimulates Energy Expenditure and Modulates Gut Microbiota in HFD-Fed Mice

**DOI:** 10.3390/md17010038

**Published:** 2019-01-09

**Authors:** Chongming Wu, Yue Zhou, Guihong Qi, Dong Liu, Xiaoxue Cao, Jiaqi Yu, Rong Zhang, Wenhan Lin, Peng Guo

**Affiliations:** 1 Pharmacology and Toxicology Research Center, Institute of Medicinal Plant Development, Chinese Academy of Medical Sciences & Peking Union Medical College, Beijing 100193, China; xiaoyuzhou5213@sina.com (Y.Z.); qi940201@163.com (G.Q.); snow20150@163.com (X.C.); yujiaqi_2018@163.com (J.Y.); 18302458685@163.com (R.Z.); 2 State Key Laboratory of Natural and Biomimetic Drugs, Peking University, Beijing 100191, China; liudong_1982@126.com

**Keywords:** asperlin, gut microbiota, obesity, marine drug, energy expenditure

## Abstract

Asperlin is a marine-derived, natural product with antifungal, anti-inflammatory and anti-atherosclerotic activities. In the present study, we showed that asperlin effectively prevented the development of obesity in high-fat diet (HFD)-fed mice. Oral administration of asperlin for 12 weeks significantly suppressed HFD-induced body weight gain and fat deposition without inhibiting food intake. Hyperlipidemia and liver steatosis were also substantially ameliorated. A respiratory metabolism monitor showed that asperlin efficiently increased energy expenditure and enhanced thermogenic gene expression in adipose tissue. Accordingly, asperlin-treated mice showed higher body temperature and were more tolerant of cold stress. Meanwhile, asperlin also increased the diversity and shifted the structure of gut microbiota. Oral administration of asperlin markedly increased the relative abundance of Bacteroidetes, leading to a higher Bacteroidetes-to-Fimicutes ratio. The HFD-induced abnormalities at both phylum and genus levels were all remarkably recovered by asperlin. These results demonstrated that asperlin is effective in preventing HFD-induced obesity and modulating gut microbiota. Its anti-obesity properties may be attributed to its effect on promoting energy expenditure.

## 1. Introduction

Obesity is a global epidemic, with more than 1.9 billion people being obese or overweight [1]. The abnormal lipid accumulation in liver and visceral fat tissues results in many health complications, such as type 2 diabetes, hyperlipidemia, nonalcoholic fatty liver disease (NAFLD) and cardiovascular diseases [2], which impose a heavy burden on national healthcare systems. The fundamental cause of obesity is a chronic imbalance of energy metabolism [3,4,5]. Therefore, restricting food intake and doing exercise are two common approaches to preventing obesity. Although food restriction is essential in preventing obesity, enhancing energy expenditure represents an alternative [6].

The human gut harbors more than 100 trillion microbes, which are collectively named gut microbiota. A huge body of evidence has proved that gut microbiota plays essential roles in the maintenance of human health [7,8]. Abnormal changes in the gut microbial community, which is called dysbiosis, are closely associated with many metabolic disorders including obesity, hyperlipidemia, diabetes and nonalcoholic fatty liver disease [9,10,11]. Therefore, gut microbiota is identified as a potential target for the treatment of many metabolic diseases. Many natural products such as metformin [12,13], cranberry [14] and ganoderma [15] extracts have been reported to exert their pharmacological properties through modulation of gut microbiota.

Asperlin is a fungal natural product which was isolated from marine-derived fungus *Aspergillus* sp. SF-5044 and exhibits antifungal and anti-inflammatory properties in vitro [16,17]. Recently, we revealed that asperlin isolated from marine-derived fungus *Aspergillus versicolor* LZD-44-03 prevented atherosclerosis through suppressing inflammation in high-fat diet (HFD)-fed ApoE^−/−^ mice [18]. In the present study, we tested the effects of *Aspergillus versicolor* LZD-44-03-derived asperlin on HFD-induced bodyweight gain, liver steatosis and energy imbalance in C57BL/c mice. We also evaluated the impact of asperlin on gut microbiota, aiming to provide insights into the mechanisms used in preventing HFD-induced obesity and liver steatosis.

## 2. Results

### 2.1. Oral Administration of Asperlin Prevents HFD-Induced Obesity

**Figure 1:**
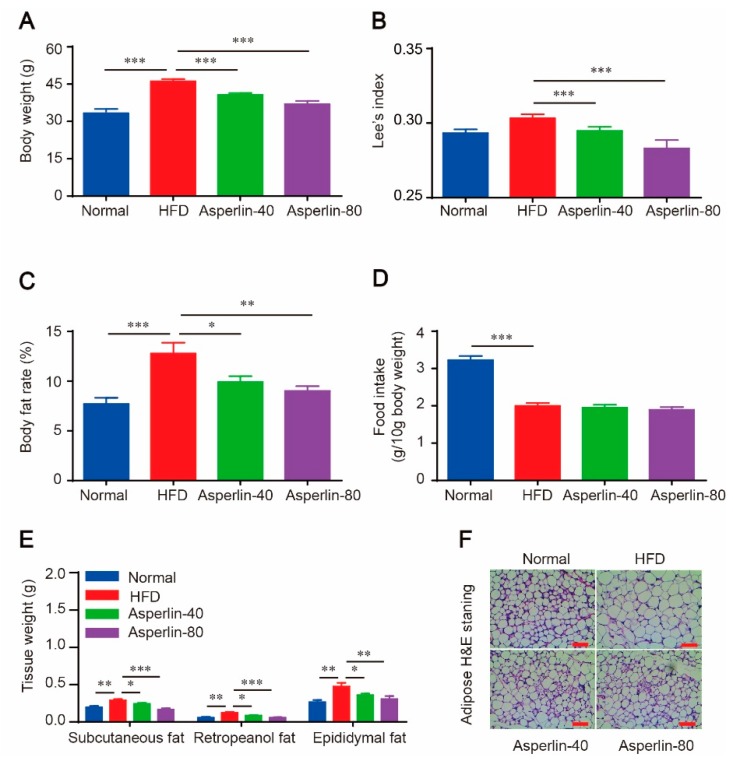
Asperlin prevents HFD-induced obesity in C57BL/c mice. (**A**) Body weight; (**B**) Lee’s index; (**C**) body fat rate; (**D**) average food intake; (**E**) tissue weights; (**F**) H&E staining of inguinal subcutaneous fat. Animals (n = 10) were fed with normal or high-fat diet and treated with asperlin (40 or 80 mg/kg) for 12 weeks. Data are shown as means ± sem. * *p* < 0.05, ** *p* < 0.01, *** *p* < 0.001 assessed by one-way ANOVA and Dunnett’s test as a post hoc test.

C57BL/6J mice were fed with a high-fat diet (HFD) for 12 weeks to induce obesity. Treatment with asperlin (40 and 80 mg/kg) noticeably suppressed body weight gain. Compared with the vehicle control (HFD group), animals given asperlin showed lower body weight, smaller Lee’s index and reduced body fat rate (Figure 1A–C). Accordingly, the weights of subcutaneous, retropeanol and epididymal fats were all markedly decreased in asperlin-treated groups (40 and 80 mg/kg) (Figure 1E). The adipocyte size in the inguinal subcutaneous fat was dramatically reduced by asperlin (Figure 1F). However, the food intake was not significantly varied between HFD and asperlin groups, indicating that the obesity-preventing effect of asperlin was not due to food restriction.

### 2.2. Asperlin Improved Serum Lipid Profile and Ameliorated Liver Steatosis

**Figure 2:**
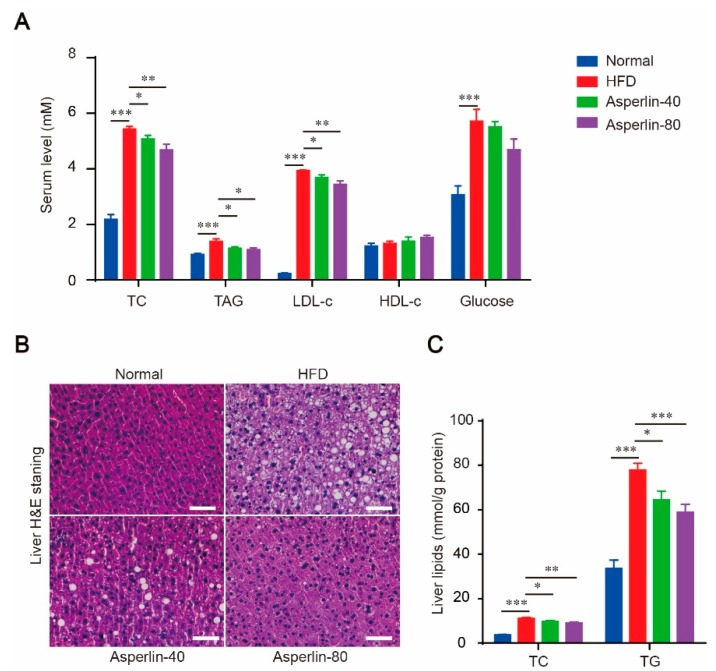
Asperlin ameliorates HFD-induced hyperlipidemia and liver steatosis. (**A**) Serum levels of lipids and glucose; (**B**) H&E staining of liver; (**C**) liver content of TC and TG. Animals (n = 10) were fed with normal or high-fat diet and treated with asperlin (40 or 80 mg/kg) for 12 weeks. Data are shown as means ± sem. * *p* < 0.05, ** *p* < 0.01, *** *p* < 0.001 assessed by one-way ANOVA and Dunnett’s test as a post hoc test. Bar = 100 μm.

Feeding with HFD for 12 weeks led to dramatic hyperlipidemia and liver steatosis, as revealed by significantly increased serum total cholesterol (TC), triglycerides (TAG) and low-density lipoprotein (LDL-c) (Figure 2A), as well as obvious fatty degeneration (ballooning) of hepatocytes (Figure 2B) and increased liver content of TC and TG (Figure 2C). Treatment with asperlin (40 and 80 mg/kg) largely reduced the blood levels of TC, TG and LDL-c (Figure 2A). The increased liver TC, TG contents and fat vacuoles were also substantially reversed (Figure 2B,C). These results suggested that oral administration of asperlin was effective in alleviating HFD-induced hyperlipidemia and liver steatosis. The HFD-induced hyperglycemia was also ameliorated by asperlin but did not reach statistical significance (Figure 2A).

### 2.3. Asperlin Enhanced Energy Expenditure

**Figure 3:**
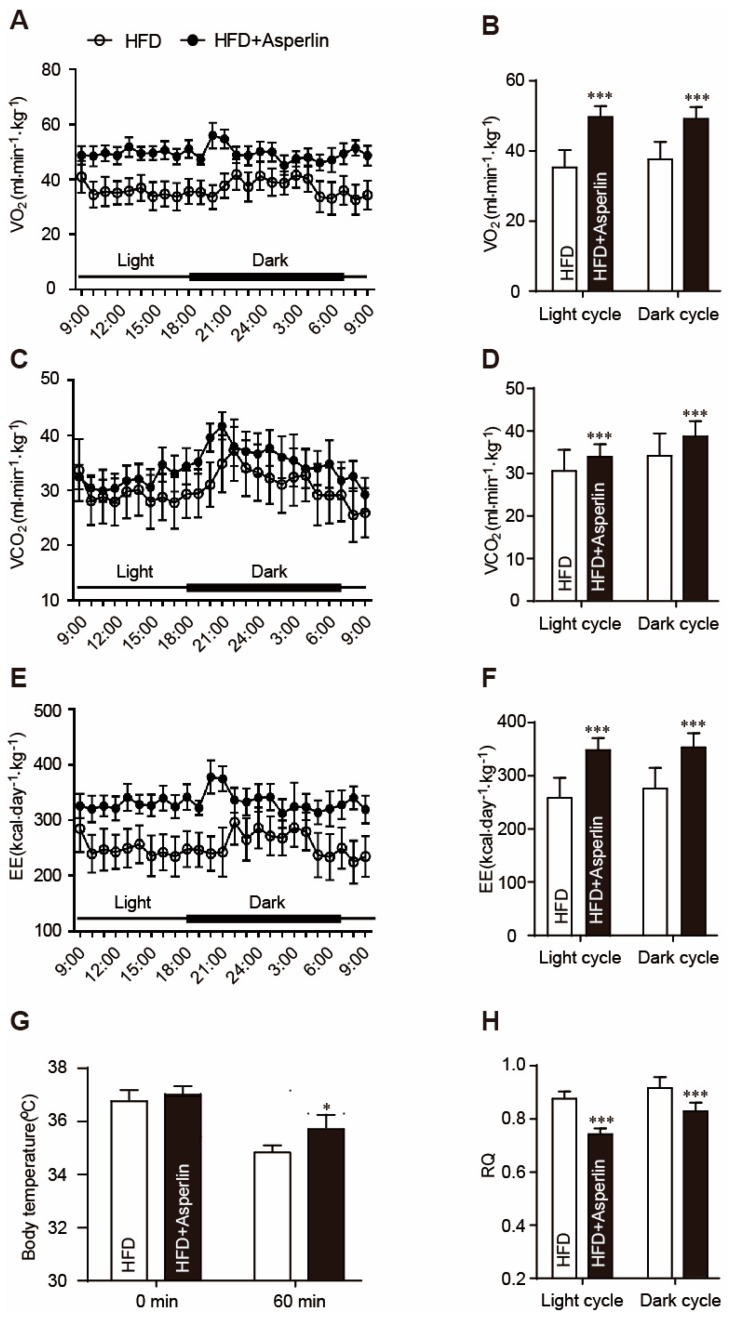
Asperlin enhances energy expenditure. (**A**,**C**,**E**) displayed the curve of oxygen consumption rate (VO_2_), carbon dioxide production rate (VCO_2_) and energy expenditure (EE) during a 24-h period, respectively. (**B**,**D**,**F**) showed that average VO_2_, VCO_2_ and EE in both light (7:00 a.m. to 6:00 p.m.) and dark (6:00 p.m. to 7:00 a.m.) cycles; (**G**) body temperature before and after cold exposure (4 °C); (**H**) the respiratory quotient (RQ) in both light and dark cycles. Animals (n = 10) were fed with high-fat diet (HFD) and treated with asperlin (80 mg/kg) for 11 weeks. The energy expenditure was monitored using CLAMS for 24 h. The body temperature was determined at the rectum before and after animals were transferred into 4 °C chamber for 1 h. Data are shown as means ± sem. * *p* < 0.05, *** *p* < 0.001 assessed by Student’s *t* test.

Increasing energy expenditure is an important way to prevent obesity [19]. We tested the effect of asperlin on animal energy expenditure by a lab animal monitoring system (CLAMS). After being normalized to bodyweight, the asperlin-treated mice displayed higher oxygen consumption rates (VO_2_), enhanced carbon dioxide production rates (VCO_2_) and increased 24-h energy expenditure (EE) (Figure 3A–F). The respiratory metabolism parameters were higher in the asperlin group (80 mg/kg) than the HFD control group at nearly every time-point. Eventually, the overall energy expenditure in both light (from 7:00 a.m. to 6:00 p.m.) and dark (from 6:00 p.m. to 7:00 a.m.) cycles were significantly increased by asperlin (Figure 3A–F). The respiratory quotient (RQ) value of asperlin-treated mice was significantly decreased. During the daytime, the RQ value in the asperlin group was around 0.7 (Figure 3H).

To further examine the effects of asperlin on energy expenditure, a cold tolerance test was performed to evaluate adaptive thermogenesis. During the one hour of cold exposure (4 °C), the body temperatures of control mice declined markedly while asperlin-treated animals showed less of a drop (Figure 3G).

**Figure 4:**
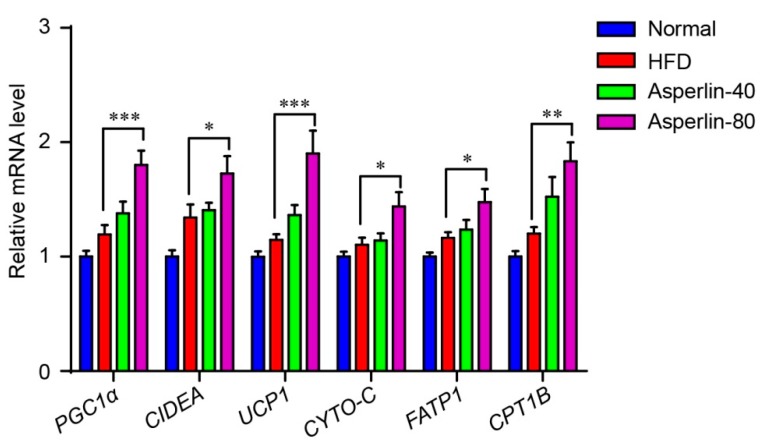
Asperlin enhanced the expression of thermogenic genes in subcutaneous fat. Animals (n = 10) were fed with normal or high-fat diet and treated with asperlin (40 or 80 mg/kg) for 12 weeks. Data are shown as means ± sem. * *p* < 0.05, ** *p* < 0.01, *** *p* < 0.001 assessed by one-way ANOVA and Dunnett’s test as a post hoc test.

We also assessed the effect of asperlin on the expression of genes that control energy expenditure and thermogenic programing of the subcutaneous fat (Figure 4). Six genes were evaluated: Peroxisome proliferator-activated receptor gamma coactivator 1-alpha (PGC1α) is a key thermogenic transcriptional factor, uncoupling protein 1 (UCP1) and cell death-inducing DNA fragmentation factor alpha-like effector A (CIDEA) are markers for adipose tissue browning, and carnitine palmitoyltransferase 1b (CPT1B), fatty acid transporter protein1 (FATP1) and cytochrome C (CYTO-C) are essential genes controlling fatty acid oxidation. The real-time PCR analysis showed that treatment with asperlin (80 mg/kg/day) significantly enhanced the transcription levels of these genes, suggesting that asperlin may promote thermogenesis through enhancing the transcription of thermogenic programming genes.

### 2.4. Asperlin Shifted the Gut Microbiota Structure

**Figure 5:**
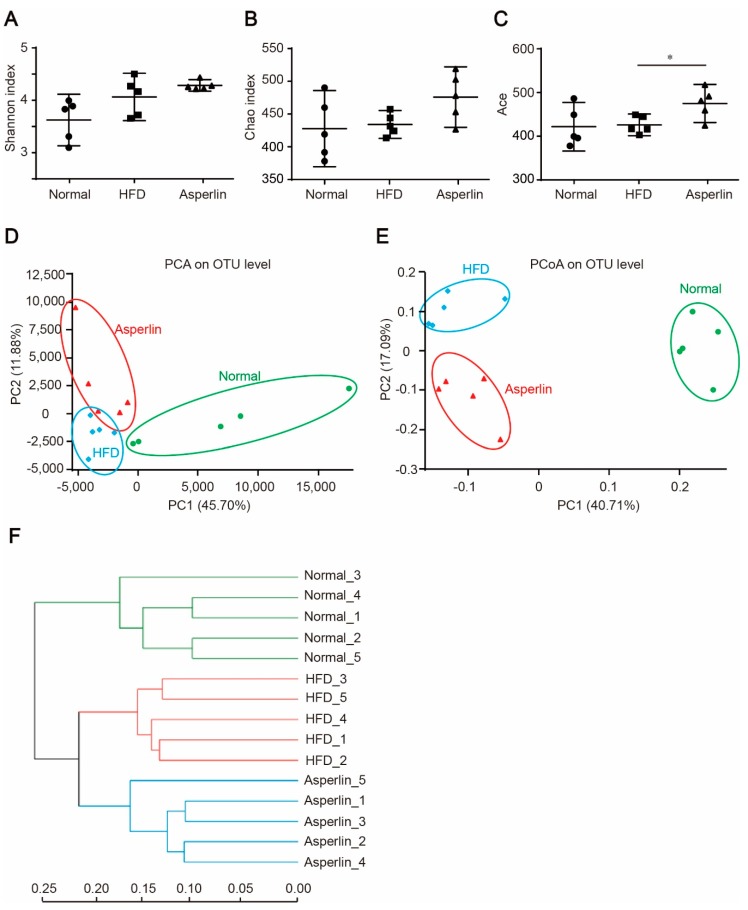
Asperlin modulates the gut microbiota structure. (**A**–**C**) displays the diversity of gut microbes as estimated by Shannon, Chao and Ace indexes. PCA (**D**) and PCoA (**E**) plots of microbial communities were based on OTU composition, each treatment group is represented by different color/symbol combinations; (**F**) average clustering of the microbial communities based on Euclidean. The asperlin group was treated with 80 mg/kg asperlin. (**A**–**C**) data are shown as means ± 95% CI. N = 5 in each group. * *p* < 0.05 assessed by one-way ANOVA and Dunnett’s test as a post hoc test.

Gut microbiota plays a key role in the development of obesity [20,21]. To explore the impact of asperlin on gut microbiota, we analyzed the composition of gut flora by 16S rRNA pyrosequencing. Compared with the vehicle control, asperlin (80 mg/kg) increased the diversity of the gut microbes as indicated by Shannon (*p* = 0.252), Chao1 (*p* = 0.066) and Ace (*p* = 0.033) indexes (Figure 5A–C). We further performed principal component analysis (PCA), principal coordinate analysis (PCoA) and UniFrac distance-based hierarchical clustering analysis on the gut microbial community. Administration of asperlin significantly shifted the structure of the gut microbiota (Figure 5D–F). Most samples in the asperlin group were completely separate from the HFD group along PC2 (*p* < 0.05) (Figure 5D,E). Hierarchical clustering analysis revealed that the asperlin-modulated gut flora were largely separate from the HFD group (Figure 5F).

### 2.5. Key Phylotypes of Gut Microbiota Responding to Asperlin

**Figure 6:**
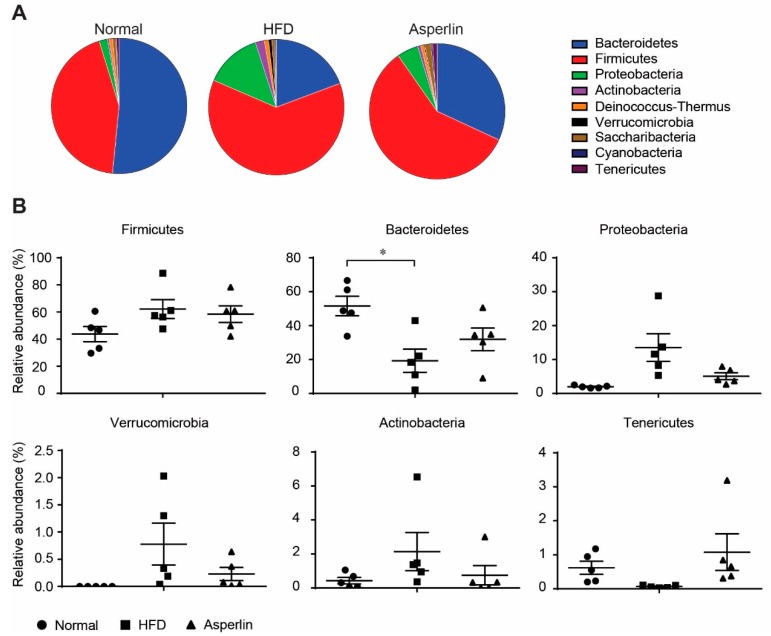
The changes of the gut microbiota structure at phylum level. (**A**) Pie chart of phylum in each group. (**B**) Scatter chart at phylum level. The asperlin group was treated with 80 mg/kg asperlin. N = 5 in each group. Data are shown as means ± sem. * *p* < 0.05 assessed by one-way ANOVA and Dunnett’s test as a post hoc test.

Taxon-based analysis also showed that asperlin markedly modulated the gut microbe community. We compared the gut microbial composition at phylum and genus levels. As shown in Figure 6, feeding with HFD led to a dramatic decrease in Bacteroidetes and a large increase in Fimicutes, which resulted in a significantly decreased Bacteroidetes-to-Fimicutes ratio (0.36 vs. 1.33, *p* = 0.031), a common change during obesity development [22]. Administration of asperlin markedly increased the relative abundance of Bacteroidetes and slightly decreased Fimicutes, increasing the Bacteroidetes-to-Fimicutes ratio, though not significantly (0.62 vs. 0.36, *p* = 0.299). In addition, oral administration of asperlin also substantially restored the abnormal changes of Proteobacteria, Verrucomicrobia, Actinobacteria and Tenericutes by HFD, shifting their relative abundance to that of the normal group (Figure 6).

**Figure 7:**
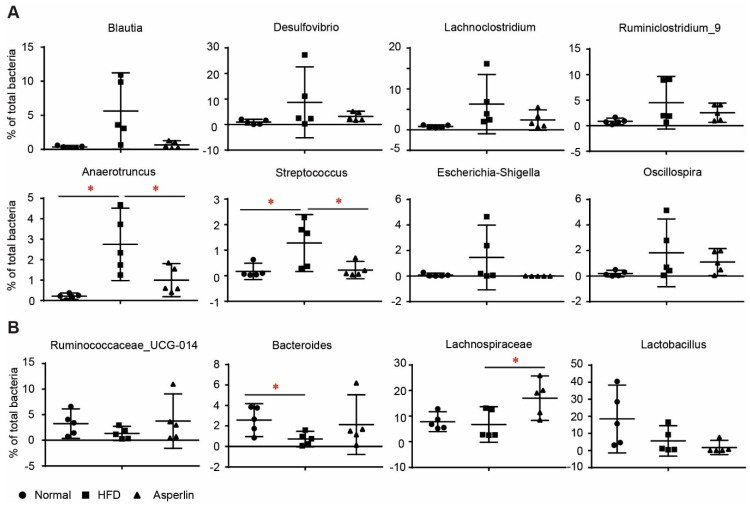
The changes to main genera by asperlin. (**A**) Genera that were increased by HFD; (**B**) genera that were decreased by HFD. The asperlin group was treated with 80 mg/kg asperlin. N = 5 in each group. Data are shown as means ± sem. * *p* < 0.05 assessed by one-way ANOVA and Dunnett’s test as a post hoc test.

Figure 7 shows the changes to the top 12 genera. HFD feeding resulted in a dramatic increase of *Blautia*, *Desulfovibrio*, *Lachnoclostridium*, *Ruminiclostridium_*9, *Anaerotruncus*, *Streptococcus*, *Escherichia-Shigella* and *Oscillospira* (Figure 7A). Oral administration of asperlin substantially recovered the changes to these genera, especially for *Anaerotruncus* and *Streptococcus*. Similarly, HFD-fed animals showed decreased abundance of *Ruminococcaceae*_UCG-014 and *Bacteroides*, a tendency which was reversed after treatment by asperlin (Figure 7B). For other genera such as *Lachnospiraceae* and *Lactobacillus*, asperlin did not alter their abundance compared to that which appeared in the normal group (Figure 7B). These results suggest that oral administration of asperlin remarkably influenced the gut microbial community and shifted the gut microbiota structure to a normal state.

## 3. Discussion

Asperlin is a marine-derived fungal product possessing multiple properties such as antitumor, anti-inflammation and anti-atherosclerosis [16,17,18]. In this study, we demonstrated for the first time that asperlin prevented HFD-induced obesity and alleviated hyperlipidemia. First, oral administration of asperlin for 12 weeks significantly prevented bodyweight gain, decreased fat deposit, and reduced body fat rate and Lee’s index. These effects were similar to those elicited by the approved anti-obesity drug, orlistat [23], as well as natural products such as cordycepin [24] and berberine [25], demonstrating potent activity against HFD-induced obesity. Simultaneously, asperlin noticably ameliorated hyperlipidemia and liver steatosis. The serum and/or liver levels of TC, TG and LDL-c and fat vacuole formation in hepatocytes were substantially reversed by asperlin. Hyperglycemia was also ameliorated, though the alleviation did not reach statistical significance. These results indicate that asperlin possesses adequate anti-obesity effects. Interestingly, oral administration of asperlin did not decrease food intake, suggesting that asperlin exerts its obesity-preventing effect through other ways, rather than food restriction.

Multiple studies have proved that increasing energy expenditure is an effective way to prevent obesity [3,4,5]. Many anti-obesity agents such as liraglutide [26], curcumin [27] and berberine [25] have been shown to enhance energy expenditure. Asperlin was effective in preventing HFD-induced obesity but did not inhibit appetite. We therefore tested the influence of asperlin on energy expenditure. The results showed that asperlin efficiently enhanced energy expenditure as revealed by increased oxygen consumption rate (VO_2_), carbon dioxide production rate (VCO_2_) and energy expenditure (EE). Simultaneously, the asperlin-treated mice showed a RQ value around 0.7 during the light cycle. The RQ indicates which macronutrients are being metabolized, with a value of 0.7 indicating lipids are being metabolized, 0.8 for proteins, and 1.0 for carbohydrates [28]. The data indicated that asperlin might drive animals to utilize lipids other than polysaccharide for thermogenesis during the daytime. In accordance with the enhanced energy expenditure, the asperlin-treated mice showed higher body temperature and were more tolerant to cold stress. These results proved that asperlin may exert an anti-obesity effect through stimulating energy expenditure.

Gut microbiota plays key roles in host energy homeostasis and the development of various diseases including obesity. As asperlin is known to possess antibiotic function [29], we think it may change the structure of gut microbiota, thus influencing host metabolism. Previous studies have shown that during the development of obesity, the relative abundance of Firmicutes is increased while Bacteroidetes population decreased, which results in a reduced Bacteroidetes-to-Firmicutes ratio [15,30,31]. At the same time, the diversity of gut microbiota is also decreased in obese animals [14,30]. We tested the impact of asperlin on the fecal bacterial community, which showed that it increased the diversity of gut microbes and markedly shifted the structure of gut microbiota. The relative abundance of Bacteroidetes was dramatically increased while the Fimicutes was slightly decreased, which resulted in a largely increased Bacteroidetes-to-Fimicutes ratio. These modulations were in good accordance with the anti-obesity effect. At the same time, asperlin substantially restored abnormal changes to specific bacteria at both phylum and genus levels, shifting their relative abundance to that of those appearing in normal animals. These data implied that modulation of gut microbiota might be another way that asperlin prevents HFD-induced metabolism disorders.

We then performed detailed analysis on the key taxon of gut microbes to detect the key taxons that were responsible for the anti-obesity effect of asperlin. *Anaerotruncus*, *Streptococcus* and *Lachnospiraceae* were significantly modulated by asperlin. As previously reported, *Anaerotruncus* is frequently accompanied by obesity and decreased by anti-obesity agents such as *Saccharomyces boulardii* [32]. *Streptococcus* comprises multiple pathogenic bacteria, and obesity is known to be a risk factor for streptococcus colonization [33]. HFD feeding dramatically increased *Anaerotruncus* and *Streptococcus,* while asperlin substantially decreased their abundance to the normal level. Oral administration of asperlin noticeably enhanced the abundance of *Lachnospiraceae*, a main short-chain fatty acid (SCFAs)-producing genus. The anti-obesity and anti-diabetes effects of SCFAs have been well-documented and many anti-obesity agents such as berberine [34] have been reported to exert their pharmacological effect through enhancing SCFA-producing bacteria. These results suggest that oral administration of asperlin contributes to shifting the gut microbiota structure to a normal state, which may play a potential role in its anti-obesity effect. However, the above data cannot distinguish whether the changes in microbiota are a cause or just a consequence of asperlin’s activity. Further causal studies are needed to clarify the importance of gut microbiota on the asperlin-mediated anti-obesity effect.

## 4. Materials and Methods

### 4.1. Asperlin Preparation

Asperlin was prepared from fungal strain *Aspergillus versicolor* LZD4403 which was isolated from gorgonian (*Pseudopterogorgia* sp.) collected from Leizhou Island in Guangdong Province of China in June 2015. The extraction, isolation and structural identification were performed as previously reported [18].

### 4.2. Animals and Experiment Design

All the animal experiments were performed in accordance with the National Institutes of Health regulations for the care and use of animals in research and were approved by the Medical Ethics Committee of the Institute of Medicinal Plant Development, Peking Union Medical College (No. YZS201711021). All efforts were made to minimize the animals’ suffering.

Forty male C57BL/6J mice, 8-weeks old, weighing 20–25 g, were purchased from Vital River Laboratory Animal Technology Co., Ltd. (Beijing, China). The animals were maintained in a humidity-controlled room on a 12 h/12 h light/dark cycle to adapt to the environment for one week. Then the mice were divided randomly into four groups (n = 10): Normal, HFD, Asperlin-40 and Asperlin-80 group. The normal group was fed with standard rodent chow which contained 5.0% fat, 75.0% carbohydrate and 20.0% protein, while other groups were given a high fat diet D12492 which contained 60.0% fat, 20% carbohydrate and 20.0% protein. The normal and HFD groups were given an equal volume of solvent (0.5% sodium carboxymethyl cellulose (CMC-Na)) while the test groups (Asperlin-40 and Asperlin-80) were administrated by oral gavage with asperlin (40 and 80 mg/kg/day respectively). The bodyweight and food intake were monitored once a week. At the 11th week after treatment, all mice underwent an energy expenditure test with a lab animal monitoring system (CLAMS). At end of the 12-week treatment, animals were fasted overnight and blood samples were collected for estimation of plasma parameters by kits. Animals were then euthanized, and the liver and fat tissues (subcutaneous fat, epididymal fat and retropeanol fat) were weighed. A bulk of the tissue was immediately frozen in liquid nitrogen for molecular and biochemical measurements. The rest was fixed in 4% paraformaldehyde for histology analysis.

Lee’s index was used as an approximate parameter for the obesity degree of each mouse. The calculation of Lee’s index was as follows: Lee’s index = bodyweight (g)^1/3^/body length (cm) [35]. The obesity degree was also characterized by the body fat rate which was measured by a minispec benchtop Time Domain NMR (TD-NMR) analyzer (Bruker Corporation, Rheinstetten, Germany). The body fat rate (%) means the percentage of fat composition in the whole body weight.

### 4.3. Metabolic Rate and Physical Activity

The energy expenditure was monitored using CLAMS (Columbus Instruments, Columbus, OH, USA). First, animals were acclimated to the system for 24 h, then the oxygen consumption rate (VO_2_), carbon dioxide production rate (VCO_2_) and physical activity of each mouse were measured in the following 24 h. The energy expenditure (EE) and respiratory quotient (RQ) were automatically calculated by the system based on VO_2_, VCO_2_ and bodyweight. All animals were kept at 24 °C. Food and water were available *ad libitum*. Voluntary activity was recorded every 15 min.

### 4.4. Cold Tolerance Test

The animals were subjected to a cold room (4 °C) without access to food or water. The body skin temperature was measured with an infrared camera (Fluke TiX660 Infrared Camera, Fluke Corporation, Everett, Washington, DC, USA) and analyzed with a specific software package (Smartview 3.14.45).

### 4.5. Histology Analysis

The fat and liver tissues of each mouse were fixed in 4% formaldehyde, embedded in paraffin and cut at 4 μm. The sections were stained with hematoxylin and eosin (H&E), and their morphological changes were evaluated. At least 20 slides of each group were analyzed.

### 4.6. Quantitative Realtime-PCR Analysis

Total RNA extraction, cDNA synthesis and quantitative polymerase chain reaction (PCR) assays were performed as described previously [36]. At least three independent biological replicates were performed to check the reproducibility of the data. The gene-specific primers used for quantitative PCR are listed in Supplementary Table S1.

### 4.7. Phylogenetic Analysis

Fecal samples were collected from each mouse, snap-frozen in liquid nitrogen, and stored at −80 °C. Fecal DNA extraction, PCR amplication and 16S rRNA pyrosequencing at the V4–V5 regions were performed by Majorbio BioTech Co., Ltd. (Shanghai, China) as previously described [37].

### 4.8. Phylogenetic Data Analysis

Metagenomic analysis as performed by R package. The alpha diversity of the microbiome was calculated based on the OTU level by mothur (version 1.30.1). Principal component analysis (PCA) and principal coordinate analysis (PCoA) were performed using R and visualized by the R package. The *p*-values were adjusted for multiple comparisons using the Benjamini and Hochberg False discovery rate and significance was set at *q* < 0.05.

### 4.9. Statistical Analyses

All data are presented as mean ± SEM. Comparisons between groups were assessed by one-way analysis of variance and Dunnett’s test as a post hoc test. A *p*-value < 0.05 was considered statistically significant.

## 5. Conclusions

In this study, we present evidence to support that the marine-derived natural product asperlin is effective in preventing high-fat diet induced obesity and modulating gut microbiota. The anti-obesity effects of asperlin may mainly be attributed to its effect on promoting energy expenditure. The results provide a potential application of asperlin in the prevention and treatment of obesity and its related complications.

## Supplementary Materials

The following are available online at http://www.mdpi.com/1660-3397/17/1/38/s1, Table S1: Oligonucleotide primers used in this work.
