# Supplementary material for: Asperlin Stimulates Energy Expenditure and Modulates Gut Microbiota in HFD-Fed Mice
Source: Mar Drugs. 2019 Jan 9;17(1):38. doi: 10.3390/md17010038 (PMC6356881; doi:10.3390/md17010038)
Supplement: Supplementary file 1 [file marinedrugs-17-00038-s001.pdf]

marine drugs

Supplementary Materials

Asperlin Stimulates Energy Expenditure and Modulates Gut Microbiota in HFD-Fed Mice

Chongming Wu [1,*,†], Yue Zhou [1,†], Guihong Qi [1], Dong Liu [2], Xiaoxue Cao [1], Jiaqi Yu [1], Rong Zhang [1], Wenhan Lin [2,*] and Peng Guo [1,*]

1 Pharmacology and Toxicology Research Center, Institute of Medicinal Plant Development, Chinese Academy of Medical Sciences & Peking Union Medical College, Beijing 100193, China; xiaoyuzhou5213@sina.com (Y.Z); qi940201@163.com (G.Q.); snow20150@163.com (X.C.); yujiaqi_2018@163.com (J.Y.); 18302458685@163.com (R.Z.)
2 State Key Laboratory of Natural and Biomimetic Drugs, Peking University, Beijing 100191, China. liudong_1982@126.com
* Correspondence: cmwu@implad.ac.cn (C.W.); whlin@bjmu.edu.cn (W.L.); pguo@implad.ac.cn (P.G.); Tel.: +86-10-5783-3235 (C.W.)
† These authors contributed equally to this work.

To evaluated the impact of asperlin (80 mg/kg/day) on the expression levels of genes that control energy expenditure and thermogenic programme in the subcutaneous fat tissue, we selected six thermogenic genes, that were, peroxisome proliferator-activated receptor gamma coactivator 1-alpha (PGC1α), uncoupling protein 1 (UCP1), cell death-inducing DNA fragmentation factor alpha-like effector A (CIDEA), carnitine palmitoyltransferase 1b (CPT1B), fatty acid transporter protein1 (FATP1) and cytochrome C (CYTO-C). The expression levels of these genes in subcutaneous fat tissue were assessed by real-time PCR analysis using the following gene-specific primers as listed in Table S1.

Table S1. Oligonucleotide primers used in this work.

Name	Forward (5′-3′)	Reverse (5′-3′)
CIDEA	TGCTCTTCTGTATCGCCCAGT	GCCGTGTTAAGGAATCTGCTG
UCP1	ACTGCCACACCTCCAGTCATT	CTTTGCCTCACTCAGGATTGG
PGC1α	AGCCGTGACCACTGACAACGAG	GCTGCATGGTTCTGAGTGCTAAG
CYTOC	CCAAATCTCCACGGTCTGTTC	ATCAGGGTATCCTCTCCCCAG
FATP1	CGCTTTCTGCGTATCGTCTG	GATGCACGGGATCGTGTCT
CPT1B	ACCACTGGCCGCATGT	CTCCATGGCGTAGTAGTTGCT
β-actin	GGCTGTATTCCCCTCCATCG	CCAGTTGGTAACAATGCCATGT
